# Study of Cardiovascular Risks in Adolescents (ERICA): alcohol consumption and associated factors

**DOI:** 10.1590/1980-549720230025

**Published:** 2023-05-08

**Authors:** Fernanda Garcia Gabira Miguez, Gabriela Oliveira, Marcia Mara Correa, Elizabete Regina Araújo Oliveira

**Affiliations:** I Universidade Federal do Espírito Santo, Public Health Program, Health Sciences Center – Vitória (ES), Brazil.; II Universidade Federal do Espírito Santo, Universitary hospital Cassiano Antônio de Moraes – Vitória (ES), Brazil.

**Keywords:** Adolescent, Alcohol consumption, Ethanol, Public health, Adolescente, Consumo de bebidas alcoólicas, Etanol, Saúde pública

## Abstract

**Objective:**

To analyze factors that influence alcohol consumption by Brazilian adolescents aged 12 to 17 years from the five macro-regions of Brazil, according to sociodemographic, schooling, and family characteristics.

**Methods:**

This is a cross-sectional study with data from the Study of Cardiovascular Risks in Adolescents (ERICA). The outcome was assessed by alcohol consumption and considered the variables sex, age, ethnicity/skin color, maternal schooling level, having housemaids, number of bathrooms at home, living or not with parents, and type of school. For the analyses, the survey mode was used for complex samples. Poisson regression was performed to assess the magnitude of factors associated with alcohol consumption among adolescents.

**Results:**

The prevalence of alcohol consumption by adolescents was 22.1%. The variables age range of 15 to 17 years, higher socioeconomic status, and living alone or with only one of the parents were factors that remained associated with alcohol consumption by adolescents regardless of their region of residence. Protective factors in alcohol consumption were associated with variables related to lower economic conditions and being of Asian or indigenous descent.

**Conclusion:**

The percentage of adolescents who consume alcohol is worrisome and must be tackled with public policies and health education. Understanding which factors are related to this situation contributes to practices and policies aimed to reduce its prevalence and damage to health.

## RESUMO

**Objetivo:**

Analisar os fatores que influenciam o consumo de álcool por adolescentes brasileiros dos 12 aos 17 anos das cinco macrorregiões do Brasil, de acordo com as características sociodemográficas, escolares e familiares.

**Métodos:**

Este é um estudo transversal com dados do Estudo de Riscos Cardiovasculares em Adolescentes (ERICA). O desfecho foi avaliado pelo consumo de álcool e foram consideradas as variáveis sexo, idade, etnia/cor da pele, nível de escolaridade materna, ter empregadas domésticas, quantidade de banheiros em casa, viver ou não com os pais e tipo de escola. Para as análises, foi utilizado o modo de inquérito para amostras complexas. A regressão de Poisson foi empregada para avaliar a magnitude dos fatores associados ao consumo de álcool entre adolescentes.

**Resultados:**

A prevalência do consumo de álcool por adolescentes foi de 22,1%. As variáveis faixa etária de 15 a 17 anos, condição socioeconômica mais elevada e viver sozinho ou com apenas um dos pais foram fatores que permaneceram associados ao consumo de álcool por adolescentes, independentemente da região de residência. Os fatores de proteção no consumo de álcool estavam associados com variáveis relacionadas a condições econômicas mais baixas e à ascendência asiática ou indígena.

**Conclusão:**

A percentagem de adolescentes que consomem álcool é preocupante e deve ser abordada com políticas públicas e educação sanitária. A compreensão dos fatores relacionados com essa situação contribui para práticas e políticas destinadas a reduzir sua prevalência e os danos para a saúde.

## INTRODUCTION

Alcohol is a licit drug of interest and is consumed by a portion of adolescents. According to the World Health Organization (WHO), 43% of adolescents over 15 years old have consumed alcohol^1^. In Brazil, the prevalence of alcohol consumption by adolescents aged 13 to 17 years was 28.1% in 2019^2^, although Brazilian legislation prohibits the sale of alcoholic beverages to children and adolescents under 18 years old^3^.

The onset of alcohol consumption is increasingly early and frequent among adolescents. Alcohol consumption may be influenced by individual factors or the social context of adolescents, such as family environment, friends, and school^4^. During this stage, adolescents undergo new discoveries and changes in the social and emotional fields^5^. Thus, alcohol consumption may increase their vulnerability, compromising their physical and mental health^6^.

The earlier the consumption, the greater the risk of adolescents becoming excessive consumers throughout life^7,8^. Moreover, early consumption increases the risk of exposure to sexually transmitted infections, traffic accidents, personality disorders, poor school performance, aggressive behavior, and unintentional injuries^9–11^.

A study shows that sociocultural factors, as well as genetics and epigenetics^11^, influence the initiation and consumption of alcohol by adolescents^4^. Environmental factors significantly affect the onset and maintenance of alcohol consumption habits^12^. Studies show that factors such as socioeconomic vulnerability, characteristics of family functioning, and being a man increase the chance of alcohol consumption^13,14^. Other studies show that families that tolerate alcohol consumption by adolescents under 18 years old contribute to increase this consumption^15^. By contrast, when families are stricter on this regard, adolescents are less likely to consume alcohol^16^.

Thus, understanding of the factors related to alcohol consumption by adolescents should extend beyond its prevalence. This study addresses alcohol consumption during adolescence, aiming to analyze sociodemographic and geographic factors that influence such consumption by Brazilian adolescents aged 12 to 17 years.

## METHODS

This study used data from the Study of Cardiovascular Risks in Adolescents (ERICA), a cross-sectional, national, and school-based study, which was performed in 2013 and 2014 to assess the prevalence of cardiovascular risk factors and metabolic syndrome in a representative sample of adolescents aged 12 to 17 years. These individuals studied in public and private schools from all Brazilian capitals and the Federal District (cities with more than 100,000 inhabitants).

Data were collected in schools with a portable individual electronic data collector, model LG GM750Q, in which adolescents answered about 100 questions about sociodemographic characteristics, occupation, physical activity, diet, tobacco use, alcohol consumption, sleep, morbidities, reproductive health, oral health, and mental health. Details on the sample design and the study data collection protocol were described in previous studies^17,18^.

The question of this investigation was: *“In the last 30 days (one month), on how many days did you have at least one glass or dose of alcohol?”.* This question was used to establish the variable alcohol consumption, since it was dichotomous.

Those who answered *“never drank alcohol”* or *“no day in the last 30 days”* were classified as non-consumers of alcohol, and those who answered *“1 or 2 days”*, *“3 to 5 days”*, *“6 to 9 days”*, *“10 to 19 days”*, *“20 to 29 days”*, or *“every day”* in the last 30 days were classified as consumers of alcohol. Adolescents who did not remember or did not answer the question were excluded from the study. Thus, in total, 71,965 adolescents participated in this research.

Sex, age (age ranges of 12 to 14 and 15 to 17 years old), ethnicity/skin color (white, black, mixed-race, or other), maternal schooling level (<8 years or ≥8 years of schooling), number of bathrooms (none, 1, or ≥2), having housemaids (none, 1, or ≥2), type of school (public or private), school area (urban or rural), and family structure (living with both parents, living with only one parent, or living alone) were sociodemographic variables.

The rate of loss of information on economic class was high (around 31%), therefore this variable was not considered in the analyses. Proxy variables for economic conditions, number of bathrooms, and having housemaids were used^19^. Thus, for the analyses, a sample of 71,965 adolescents, who properly answered the question of interest, was used.

Data analyses were performed with the Stata 16.0 statistical package, using the survey mode to analyze complex sample data. Prevalence estimates were presented in proportions (%), with their respective 95% confidence intervals (95%CI). The chi-square test was used to assess the difference between prevalence rates of alcohol consumption according to demographic, socioeconomic, and school factors. To analyze possible associations between independent and dependent variables, logistic regression analysis was used. Independent variables associated with the outcome and with a significance level of p≤0.20 were analyzed by multivariate Poisson regression at the same level. They were excluded by the stepwise backward method. For all statistical analyses, a probability level of significance of 5% was considered.

This study was approved by the Research Ethics Committees of the institution of the study coordination center (IESC/UFRJ — Process 45/2008) and the 27 research institutions participating as representatives of Brazilian states. To participate in the study, all adolescents signed an agreement term and, when required by the local Research Ethics Committee, parents or guardians signed an informed consent form.

## RESULTS

Our study used data from the 71,965 adolescents selected from the 102,327 that were eligible for this study. The prevalence of alcohol consumption among adolescents was 22.1% (95%CI 21.21–23.2). Table 1 shows that Southern Brazil was the macro-region with the highest prevalence (28.7%), while Northern Brazil had the lowest (15.4%).

**Table 1:** Prevalence of consumption and non-consumption of alcohol by adolescents, Brazil and its macro-regions. ERICA, 2013–2014.

Macro-region	Consumption of alcohol			Non-consumption of alcohol		
	n	%	95%CI	n	%	95%CI
Brazil	15,611	22.1	21.1–23.2	56,354	77.9	76.8–78.9
Central West	2,195	24.3	22.3–26.3	7,175	75.7	73.7–77.6
Northeast	4,052	17.2	15.7–18.9	18,347	82.7	81.1–84.3
North	2,311	15.4	14.4–16.5	12,246	84.5	83.5–85.6
Southeast	4,102	23.4	21.6–25.4	12,353	76.5	74.6–78.4
South	2,951	28.7	26.5–31.0	6,233	71.3	70.0–73.4

p-value <0.001

Table 2 shows the profile of adolescents who consumed alcoholic beverages, according to sociodemographic variables. The highest prevalence of alcohol consumption was among Brazilian adolescents aged 15 to 17 years (30.8%; 95%CI 29.0–32.8); however, living with both parents was one of the main factors responsible for the lowest prevalence of alcohol consumption (19.8%; 95%CI 18.7–20.8).

**Table 2:** Profile of adolescents who consumed alcoholic beverages, according to sociodemographic variables, Brazil and its macro-regions. ERICA, 2013–2014.

Variables		Brazil		Central West		Northeast		North		Southeast		South	
		%	95%CI	%	95%CI	%	95%CI	%	95%CI	%	95%CI	%	95%CI
Sex													
	Girls	22.4	21.2–23.7	24.4	22.6–26.3	17.1	15.7–18.7	15.5	14.3–16.8	23.7	21.6–26.1	29.9	26.7–33.5
	Boys	21.8	20.3–23.4	24.1	21.4–27.1	17.4	15.3–19.7	15.3	14.0–16.7	23.1	20.4–26.0	27.5	24.4–30.7
Age (years old)													
	12–14	14.2	21.6–26.4	14.4	12.9–16.0	10.8	9.2–12.7	9.5	8.6–10.6	15	13.5–16.7	20.3	18.1–22.6
	15–17	30.8	29.0–32.8	35.1	31.7–8.6	24.2	22.2–26.5	22	20.4–23.6	32.8	29.4–36.4	38.4	34.6–42.4
Ethnicity													
	White	22.6	21.0–24.3	23.3	20.8–26.1	16.9	14.5–19.7	14.7	13.1–16.3	22.8	20.1–25.7	28.1	25.7–30.8
	Black	24.5	21.9–27.5	28.7	23.9–33.8	21.3	16.0–27.9	13.5	10.8–16.6	25.4	21.2–30.1	35.5	30.2–41.3
	Mixed-race	21.5	20.5–22.7	24.1	21.5–26.9	17	15.4–18.6	16	14.5–17.4	23.8	21.8–25.8	29.5	26.0–33.3
	Other	19	16.5–21.9	26.9	21.5–33.3	13.9	10.9–17.6	14	10.2–18.8	23.7	18.7–29.6	14.1	9.1–21.1
Number of bathrooms													
	None	14.6	7.8–25.6	65.7	25.9–91.3	6.7	2.7–15.7	21.3	12.4–34.1	6.8	1.6–24.8	22.2	5.2–59.8
	1	21.3	20.3–22.4	23.4	20.7–26.5	17	15.0–19.1	14.4	13.2–15.7	22.7	21.1–24.4	27	24.3–29.8
	≥2	23.3	21.7–25.0	24.8	22.5–27.3	17.8	15.9–19.7	16.8	15.3–18.4	24.5	21.5–27.8	31.2	28.8–33.7
Housemaids													
	None	21.3	20.2–22.4	23.1	20.9–25.4	16.7	14.9–18.6	14.6	13.5–15.7	22.5	20.6–245	28	25.8–30.4
	1	24.6	22.2–27.2	26.6	22.6–31.0	19.3	16.7–22.1	20.1	17.1–23.5	26.2	21.6–31.3	30.2	25.9–34.8
	≥2	29.6	26.8–32.4	32.4	26.4–38.9	20.6	15.8–26.2	17.9	14.4–21.9	32.5	28.1–37.4	39.4	33.1–46.1
Maternal schooling level													
	<8	21.8	19.9–23.8	25.7	22.1–29.8	15.8	13.3–18.7	15.4	13.1–18.0	23.4	20.1–27.1	28.4	24.7–32.3
	≥8	22.5	21.3–23.8	24.1	21.7–26.5	18.2	16.4–20.1	15.1	13.9–16.2	23.7	21.7–25.9	29.7	26.5–33.0
Family structure													
	Lives with both parents	19.8	18.7–20.8	20.3	18.2–22.6	15.5	13.7–17.5	13.4	12.2–14.7	20.8	19.1–22.6	25.9	23.3–28.7
	Lives with only one parent	24.6	23.2–26.0	29.3	26.1–32.7	19.1	17.2–21.2	17.2	15.7–18.9	25.7	23.2–28.3	33.2	30.8–35.7
	Lives alone	30	25.4–35.1	28.4	23.6–33.8	19.9	16.9–23.2	18.1	15.2–21.4	41.3	31.3–51.9	29.9	23.9–36.7
Type of school													
	Public	22.2	20.9–23.3	24.3	22.1–26.6	17.9	15.9–20.2	15.2	14.1–16.4	23.3	21.3–25.4	28.1	25.9–30.4
	Private	21.1	18.6–25.9	24.1	19.1–29.8	14.9	12.1–18.1	17.3	12.5–23.5	24.1	18.1–31.2	32.7	24.2–42.4
School area													
	Urban	22.1	21.0–23.1	24.2	22.4–26.5	16.9	15.3–18.7	15.5	14.4–16.6	23.3	21.4–25.3	29	26.8–31.4
	Rural	24	20.4–28.1	18.3	14.2–23.4	24.9	17.1–34.7	12.6	8.8–17.5	26.3	22.6–30.4	11.2	3.9–28.3

Table 2 also shows that, among the five macro-regions of Brazil, adolescents aged 15 to 17 years presented the highest prevalence of alcohol consumption and Southern Brazil was the macro-region with the highest prevalence (38.4%; 95%CI 34.6–42.4). Regarding family structure, the highest prevalence of alcohol consumption was among adolescents who lived alone in comparison with those who lived with their parents. In Southeastern Brazil, the highest prevalence of alcohol consumption was among adolescents who lived without their parents (41.3%; 95%CI 31.3–51.9).

The logistic regression crude analysis of the five macro-regions of Brazil and the country as a whole are included in the supplementary material, where adolescents aged 15 to 17 years, who had more than two bathrooms, housemaids (factors associated with better economic conditions), and lived without their parents presented the highest chances of alcohol consumption).

A multivariable-adjusted analysis (Table 3) showed that, in Brazil, the age range of 15 to 17 years was associated with a higher alcohol consumption: these adolescents had more than doubled the chance of consuming this kind of beverage (odds ratio — OR 2.64; 95%CI 2.36–2.94). Having housemaids (OR 1.54; 95%CI 1.03–1.22) and a higher number of bathrooms (OR 1.12; 95%CI 1.03–1.22), which were proxy variables for better economic conditions, were also associated with alcohol consumption by adolescents. In our study, adolescents who lived in houses without bathrooms — a proxy variable for lower economic conditions — had lower chances of alcohol consumption (OR 0.54; 95%CI 0.31–0.95). The chance of alcohol consumption by adolescents of Asian or indigenous descent was 24% lower in comparison with other ethnicities/skin colors (OR 0.76; 95%CI 0.62–0.93). Regarding family structure, living with only their mother or father (OR 1.34; 95%CI 1.22–1.46) or living alone (OR 1.71; 95%CI 1.34–2.17) also increased the chance of alcohol consumption by adolescents.

**Table 3:** Sociodemographic factors associated with alcohol consumption by adolescents. Adjusted analysis of Brazil and its macro-regions. ERICA, 2013–2014.

Variables		Brazil		Central West		Northeast		North		Southeast		South	
		OR	95%CI	OR	95%CI	OR	95%CI	OR	95%CI	OR	95%CI	OR	95%CI
			(p-value)		(p-value)		(p-value)		(p-value)		(p-value)		(p-value)
Age													
	15–17	2,64	2,36–2,94	3,15	2,65–3,75	2,69	2,28–3,16	2,65	2,35–2,99	2,76	2,27–3,35	2,47	2,03–2,99
			<0,001		<0,001		<0,001		<0,001		<0,001		<0,001
Ethnicity													
	Black	–	–	–	–	–	–	–	–	–	–	1,43	1,02–2,01
													<0,035
	Mixed-race	–	–	–	–	–	–	–	–	–	–	–	–
	Other	0,76	0,62–0,93	–	–	–	–	–	–	–	–	0,33	0,16–0,66
			<0,008										<0,002
Number of bathrooms													
	None	0,54	0,31–0,95	–	–	–	–	–	–	–	–	–	–
			<0,034										
	≥2	1,12	1,03–1,22	–	–	–	–	–	–	–	–	1,31	1,15–1,49
			<0,007										<0,001
Housemaids													
	1	1,26	1,13–1,40	1,45	1,17–1,80	1,32	1,09–1,59	1,5	1,24–1,82	1,33	1,11–1,59	–	–
			<0,008		<0,001		<0,003		<0,001		<0,002		
	≥2	1,54	1,34–1,78	1,53	1,16 – 2,18	1,37	1,00 – 1,89	–	–	1,72	1,36–2,16	1,79	1,29–2,48
			<0,001		<0,01		<0,05				<0,001		<0,001
Family structure													
	Lives with father or mother	1,34	1,22–1,46	1,55	1,28–1,86	1,24	1,11–1,49	1,31	1,15–1,48	1,3	1,11–1,52	1,5	1,30–1,74
			<0,001		<0,001		<0,002		<0,001		<0,001		<0,001
	Lives without father and mother	1,71	1,34–2,17	1,4	1,10–1,79	–	–	1,32	1,06–1,65	2,75	1,73–4,36	–	–
			<0,001		<0,006				<0,013		<0,001		

In Central-Western Brazil (Table 3), being 15 to 17 years old (OR 3.15; 95%CI 2.65–3.75), having housemaids (OR 1.53; CI95% 1.16–2.18), living with only the father or mother (OR 1.55; 95%CI 1.28–1.86), or living alone (OR 1.40; 95%CI 1.10–1.79) were factors that increased the chance of alcohol consumption by adolescents. In Northeastern Brazil, being 15 to 17 years old increased the chance of alcohol consumption by adolescents by almost three times (OR 2.69; 95%CI 2.28–3.16).

Having housemaids and living with only the father or mother were also associated with alcohol consumption (OR 1.32; 95%CI 1.09–1.59 and OR 1.24; 95%CI 1.11–1.49, respectively), which is similar to the results for Northern Brazil. In Southern Brazil, being 15 to 17 years old, being black, having a greater number of bathrooms, having housemaids, and living with only the father or mother were factors associated with a higher alcohol consumption by adolescents.

## DISCUSSION

This study estimated the prevalence of alcohol consumption and evaluated its association with the socioeconomic status of Brazilian adolescents. The general prevalence of alcohol consumption was close to that found in the National Survey of School Health (PeNSE) 2019^2^ and in the 3^rd^ National Survey on Drug Use by the Brazilian Population^20^. Differences in prevalence, according to the macro-region, ranged from 15.4% (Northern Brazil) to 28.7% (Southern Brazil), which is in accordance with data from the PeNSE 2015 and 2019^2,21^.

The prevalence was similar between girls and boys (22.4 and 21.8%, respectively), showing that alcohol consumption was similar regardless of sex, which is in accordance with a study by Strauch et al.^8^. However, the PeNSE 2019 found a prevalence of alcohol consumption of 30.1% among girls and 26% among boys^2^. Several studies have observed a higher prevalence among women^21–23^.

Adolescents aged 15 to 17 years showed a higher prevalence of alcohol consumption both in Brazil (30.8%) and its macro-regions, both in the crude and adjusted analyses. Finding friends and identifying with a specific group is particularly important at this age^24^ and alcohol consumption is seen as a facilitator of socialization, as well as a way to satisfy curiosity about the effect of alcohol^24^. Moreover, high exposure to advertisements of this product makes that specific public, which seeks fun, the company of friends, and an escape from reality, create expectations^24^.

This study used a proxy for socioeconomic status with the variables number of bathrooms and having housemaids. Our results suggest that better living conditions (greater number of bathrooms and having housemaids) favor alcohol consumption. In Brazil and its five macro-regions, having more than two bathrooms and housemaids increased the chances of alcohol consumption by adolescents.

These results corroborate other studies^25,26^. In the United States, a longitudinal survey with national data showed that a higher income of parents is associated with alcohol consumption and the use of other drugs (25%). For the author, adolescents with better economic conditions have more opportunity to consume alcohol in comparison with adolescents with lower economic conditions^27^. A study shows that adolescents with higher household incomes can buy alcohol more easily and in sufficient quantity even to get drunk.

The type of school (public or private) may reflect socioeconomic differences of adolescents, influencing exposure to alcohol. However, our findings showed no statistically significant difference between adolescents who studied in public and private schools regarding alcohol consumption, which corroborates the results of the PeNSE 2019: the type of school did not influence the prevalence of alcohol consumption by adolescents^2^.

Only in Southern Brazil was ethnicity/skin color associated with increased alcohol consumption by adolescents. Being black increased by 1.4 times the chance of alcohol consumption by adolescents in this macro-region (95%CI 1.02–2.01). A study by Jomar et al., performed in the state of Rio de Janeiro, Brazil, found a higher chance of alcohol consumption among black people in comparison with white people (OR 4.83), although their study was performed with adults^28^. Our findings differ from other studies performed in Brazil regarding the stronger association of ethnicity/skin color with alcohol consumption by adolescents. The PeNSE^2^ showed a higher prevalence of alcohol consumption among white than among black adolescents. More research is recommended in order to elucidate possible reasons for the higher alcohol consumption by black people in Southern Brazil.

In this study, family structure was associated with alcohol consumption by adolescents, as living alone or with only one of their parents increased the chance of consumption (OR 1.71; 95%CI 1.34–2.17 and OR 1.34; 95%CI 1.22–1.46, respectively). Lack of parental supervision has a dose-response relationship regarding alcohol consumption; thus, the lower the parental supervision, the greater the chance of alcohol consumption by adolescents^29^. The results of the PeNSE 2015 showed that having a parent who consumes alcohol increases the chance of alcohol consumption by adolescents (OR 3.21 and OR 2.14, respectively)^30^.

A dysfunctional family dynamic, with frequent discussions between the adolescents and their parents, lack of dialogue, and parents’ lack of participation in their children's daily activities enhances exposure to risk situations^31^, such as alcohol consumption. Several studies show that having a family member who uses any kind of drug (legal or illegal) influences its consumption by adolescents^7,31,32^.

On the other hand, in an environment of family support, adolescents who put their family's needs above their own tend to consume less alcohol^19^. The presence of parents has a direct relationship with discipline and rules, which leads adolescents to develop social skills, choose better friends, and recognize risks^33^. Parental supervision is extremely relevant in the prevention of health risk factors, especially alcohol consumption.

Despite the prohibition by Brazilian legislation^3^, since 2015, of selling, supplying, serving, administering, or delivering alcoholic beverages to children or adolescents, even if for free, is punishable by two to four years of prison and a fine. This increases the need for monitoring, awareness, and policies aimed to reduce the contact of young people with alcohol. In supermarkets, for example, alcoholic beverages are exhibited in areas of easy access and visibility. The short-, medium-, and long-term measures of the current Brazilian legislation are expected to be applied all over the country, in order to protect children and adolescents from damage caused by abusive alcohol consumption.

More severe damage leading to mental problems has been described as relevant not only in Brazil's public health system, but worldwide, showing that alcohol consumption by adolescents can have a deleterious effect on health^34^. Moreover, alcohol increases the risk of cardiovascular diseases, such as systemic arterial hypertension (SAH), coronary artery disease (CAD), and cerebrovascular accident (CVA)^35,36^.

As a measure to reduce damage, the Brazilian Ministry of Health regulated the Psychosocial Care Centers Alcohol and Other Drugs (CAPS-AD)^37^ and implemented the National Policy on Integral Attention to the Health of the Child (PNAISC)^38^ to recognize the rights of children and adolescents in cases of abuse of alcohol and other drugs in the family, understanding the importance of treatment for the integral health of this group. In 2019, the WHO released the SAFER technical package, which provides support to the member states for reducing harmful alcohol consumption by strengthening actions to reduce this consumption^5^.

This study has limitations. First, there is an information bias regarding alcohol consumption, since it may have prevented some adolescents from revealing their actual habits, even in anonymity, due to censorship, lack of memory, guilt, or because they were in the school environment. Secondly, much data was lost in the assessment of economic level. Thus, variables that showed the economic class of adolescents, such as having housemaids and number of bathrooms, only suggest that they belong to higher economic classes.

Therefore, scientific production on factors associated with alcohol consumption by adolescents in Brazil is indispensable. It is a complex and multifactorial issue, with major consequences to the health of a vulnerable group. Understanding which factors influence alcohol consumption will contribute to implement health education within both the school and family. Public policies with strategies aimed to promote adolescent health, resulting in changes in lifestyle and consequently reducing the early consumption of psychoactive substances are of great relevance and should be considered in the field of health.
